# EPR spectroscopy reveals different Cu(ii) coordination in APP_142–172_ and APP_145–170_ peptide fragments of amyloid precursor protein

**DOI:** 10.1039/d6ra00647g

**Published:** 2026-03-19

**Authors:** Liya Xu, Jian Kuang, Aokun Liu, Lianghuan Liao, Lu Yu, Changlin Tian

**Affiliations:** a High Magnetic Field Laboratory, Hefei Institutes of Physical Science, Chinese Academy of Sciences Hefei Anhui 230031 China cltian@ustc.edu.cn; b Department of Environmental Science and Engineering, University of Science and Technology of China Hefei Anhui 230026 China luyuesr@ustc.edu.cn; c School of Chemistry and Chemical Engineering, Zhang Jiang Institute for Advanced Sciences, Shanghai Jiao Tong University Shanghai 201203 China; d Beijing Life Science Academy Beijing 102206 China

## Abstract

Amyloid precursor protein (APP) is central to Alzheimer's disease pathogenesis, yet the coordination chemistry and functional impact of core peptide fragments within its copper binding domain (CuBD) remain elusive. Here, we characterised the copper coordination environments and redox properties of two CuBD fragments APP_142–172_ and APP_145–170_ using electron paramagnetic resonance (EPR) and UV-Vis spectroscopy. At physiological pH, Cu(ii)–APP_142–172_ adopted a single N_2_O_2_ coordination, whereas Cu(ii)–APP_145–170_ existed in two distinct coordination modes identified by spectral simulation: the same N_2_O_2_ form (component I) as in Cu(ii)–APP_142–172_, and a nitrogen-rich 4N form (component II). Moreover, EPR-monitored pH titrations revealed that the 4N species predominated at alkaline pH and the N_2_O_2_ species at acidic pH. Although both Cu(ii)–APP complexes could promote Fe(ii) oxidation, only the N_2_O_2_ species (component I) exhibited ferroxidase activity, whereas the 4N species (component II) was redox-silent. These observations demonstrate that subtle changes in peptide length act as a structural switch for Cu(ii) coordination and redox activity, thereby affecting the copper-mediated regulation of neuronal redox processes.

## Abstract 2

Subtle APP sequence variations influence copper coordination: APP_142–172_ adopts redox-active N_2_O_2_ configuration while APP_145–170_ exhibits both redox-active N_2_O_2_ and redox-silent 4N modes.

## Graphical abstract

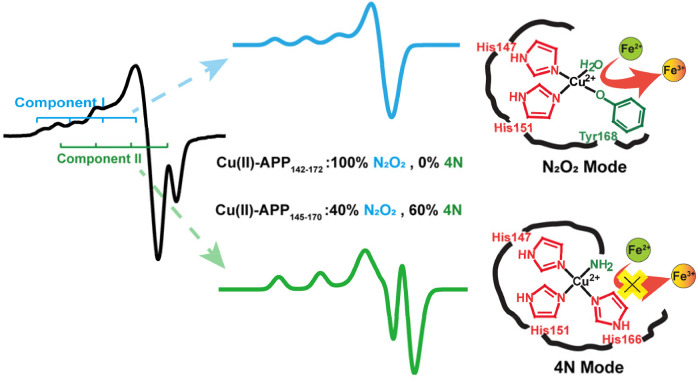

## 1. Introduction

Copper is an essential trace element in the brain, participating in numerous biochemical processes, including neuronal metabolism, energy production, and neurotransmitter synthesis.^1,2^ Disruption of copper homeostasis can induce oxidative stress, neuroinflammation, mitochondrial dysfunction, and abnormal protein aggregation or misfolding, ultimately accelerating the progression of neurodegenerative diseases.^3^

Amyloid precursor protein (APP) plays a central role in Alzheimer's disease (AD). During cellular processing, APP is cleaved to produce β-amyloid (Aβ), the major component of the characteristic plaques found in AD brains.^4,5^ APP contains an extracellular copper-binding domain (CuBD) that binds Cu(ii) with nanomolar affinity and exhibits redox activity.^6,7^ High-resolution structural studies have identified His147, His151, and Tyr168 as the principal coordinating residues within the CuBD, whereas Met170 may participate in electron transfer.^8,9^

Previous studies have predominantly examined either isolated binding motifs or full-length domains; however, few have directly compared highly similar peptide fragments under controlled, identical conditions. Such comparisons are especially lacking for fragments containing the CuBD core sequence, which incorporates both coordinating and potentially redox-active residues. Consequently, how subtle sequence variations and solution pH influence copper coordination geometry, species distribution, and complex stability remains to be elucidated. Recent spectroscopic and computational studies further reinforced that Cu(ii) binding to APP- and Aβ-derived sequences occurs as a dynamic ensemble of coexisting coordination modes, with populations that are highly sensitive to local sequence, pH, and steric environment.^10,11^

To address this gap, we selected two peptide fragments derived from the CuBD core region: the longer APP_142–172_ and the shorter APP_145–170_. Both fragments incorporate essential coordinating residues and Met170, thereby possessing dual copper-coordination and redox functionalities. Electron paramagnetic resonance (EPR) and UV-Vis spectroscopy were combined with pH titration to resolve coordination states and determine species distributions. Redox properties were evaluated by monitoring the oxidation of Fe(ii). EPR probes the unpaired electrons of Cu(ii), revealing ligand types and the electronic states of metal centre, thereby enabling the identification of distinct coordination modes and monitoring of electron transfer during redox processes.^12,13^

## 2. Results and discussion

**Fig. 1:**
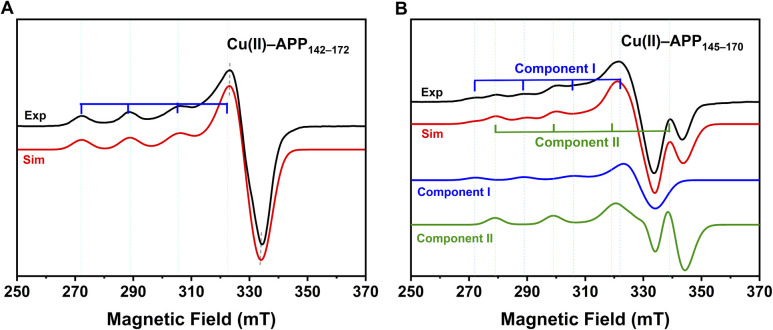
Experimental and simulated X-band CW-EPR spectra of the two copper–peptide complexes at pH 7.4. (A) Cu(ii)–APP_142–172_. (B) Cu(ii)–APP_145–170_. Black traces: Experimental spectra. Red traces: simulated spectra. In panel (B), the blue and green traces represent the individual simulated components (component I and component II), displayed at their fitted proportions as determined by EasySpin fitting (Table S1). Residual spectra are provided in Fig. S6.

The coordination modes of Cu(ii) in the two peptide fragments were characterised using X-band EPR. In frozen buffer solution, the continuous-wave (CW) EPR spectra of both copper–peptide complexes exhibited *g* factors (*g*_∥_ > 2.1 > *g*_⊥_ > 2.00) and hyperfine splitting constants (*A*_∥_ > 120 × 10^−4^ cm^−1^), which were characteristic of typical axial coordination geometry for Cu(ii).^14^ These parameters indicated that the unpaired electron predominantly occupied the d_*x*^2^−*y*^2^_ orbital, suggesting that Cu(ii) predominantly adopted a square-planar coordination geometry. The experimental spectra of both complexes were simulated to extract *g* values and hyperfine splitting constants, as detailed in Table S1 and illustrated in Fig. 1.^15^

For Cu(ii)–APP_142–172_, the *g* factor (*g*_∥_ = 2.2725) and hyperfine constant (*A*_∥_ = 172 × 10^−4^ cm^−1^) were consistent with the characteristic features of a Type II copper centre.^16^ The spin Hamiltonian parameters placed this species in the N_2_O_2_ region of the Peisach–Blumberg plot (Fig. S5).^17^ The *g*_∥_ value of 2.2725 was higher than the 2.25 typically observed for N_2_O_2_ systems and may indicate a distortion of the square-planar geometry of the equatorial ligands toward a more tetrahedral configuration.^18^ In contrast, the Cu(ii)–APP_145–170_ spectrum was more complex, consisting of two components under physiological pH conditions (pH 7.4). As shown in Fig. 1B, component I exhibited spectroscopic features resembling those of Cu(ii)–APP_142–172_, indicating a similar mixed N/O coordination environment. The smaller *g*_∥_ value (*g*_∥_ = 2.1850) and larger *A*_∥_ value (*A*_∥_ = 200 × 10^−4^ cm^−1^) of component II suggested a nitrogen-dominated, near-square-planar coordination environment with highly localized electron density. This assignment was supported by the location of the spin Hamiltonian parameters, within the 4N region of the Peisach–Blumberg plot (Fig. S5).^17^ Furthermore, the empirical ratio *f* (*g*_∥_/*A*_∥_), commonly used as a measure of geometric configuration and deviation from ideal square planarity in Cu(ii) complexes, revealed that component II (*f* = 109 cm) was closer to an ideal square planar geometry (*f* = 105–120 cm) than component I (*f* = 132 cm) (Table S1).^19,20^ Preliminary W-band Davies ENDOR spectra (Fig. S7) further supported the involvement of nitrogen ligands in both complexes. Results from fitting based on EasySpin indicated that the nitrogen-rich, near-square-planar Cu(ii) coordination structure of component II predominated in the Cu(ii)–APP_145–170_ system (≈60%).

**Fig. 2:**
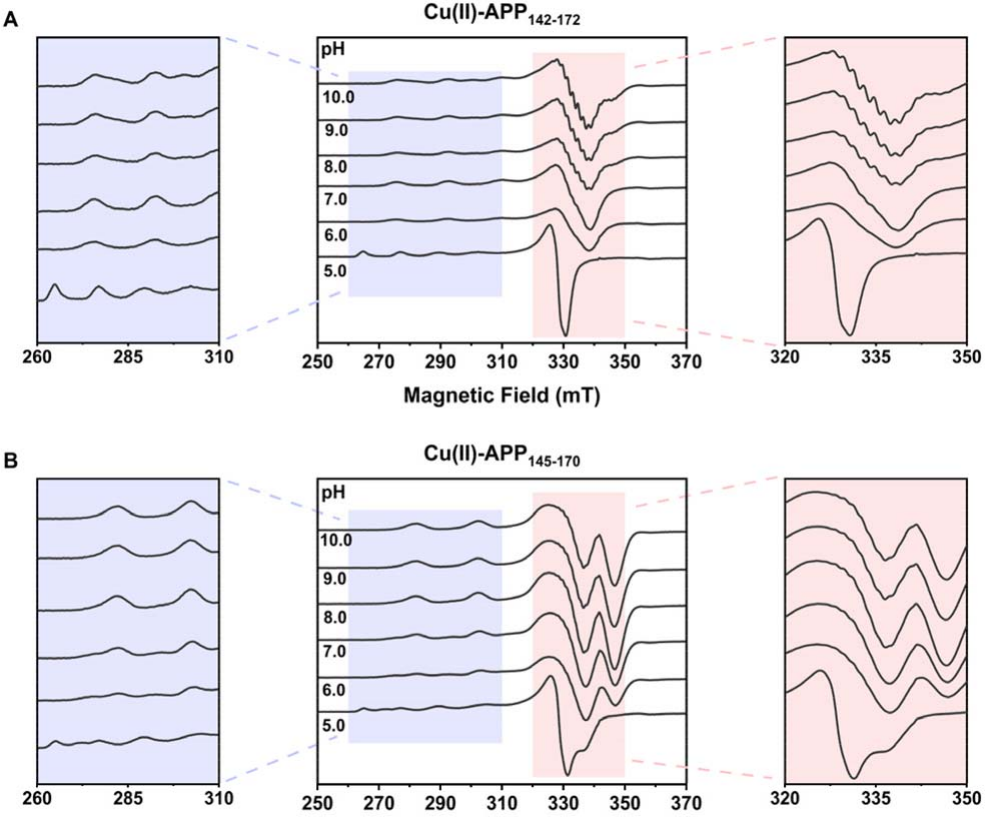
EPR spectra of Cu(ii)–APP_142–172_ (A) and Cu(ii)–APP_145–170_ (B) across pH 5.0–10.0. The spectral regions between 260–310 mT (left panels) and 320–350 mT (right panels) were expanded to highlight pH-dependent spectral differences.

Solution conditions, particularly pH, critically govern the donor availability and the coordination geometry of copper–peptide complexes. To elucidate the pH-dependent modulation of these two distinct coordination modes, EPR spectroscopy was performed over a pH range of 5.0–10.0. For Cu(ii)–APP_142–172_, as shown in Fig. 2A, the *g*_∥_ resonance at pH 5.0 resolved into four distinct hyperfine lines characteristic of the hexaaquacopper(ii) complex, [Cu(H_2_O)_6_]^2+^.^14^ This was characterized by a high *g*_∥_ value of 2.407 and a small *A*_∥_ of 135 × 10^−4^ cm^−1^ (Table S2), indicating a predominantly oxygen-based coordination environment consistent with unbound, solvated Cu(ii) under acidic conditions. With increasing pH to 6.0, *g*_∥_ decreased to 2.262 while *A*_∥_ increased to 182 × 10^−4^ cm^−1^, reflecting the progressive replacement of oxygen donors by nitrogen ligands with higher electron-donating capacity. Between pH 6.0 and 10.0, the EPR parameters remained relatively stable (*g*_∥_ ≈ 2.26, *A*_∥_ ≈ 182 × 10^−4^ cm^−1^), falling within the range expected for N_2_O_2_ coordination mode. At pH 8–10, distinguishable hyperfine structures emerged in the *g*_⊥_ region (320–350 mT, see the enlarged panel on the right of Fig. 2A), with an average splitting of ∼17 G, which was characteristic of superhyperfine interactions with ^14^N nuclei, indicating increased nitrogen participation in the coordination sphere.^21^

Cu(ii)–APP_145–170_ exhibited component-specific pH sensitivity with distinct EPR responses. Under acidic conditions (pH 5.0), the system was dominated by solvated Cu(ii) (*g*_∥_ ≈ 2.406, *A*_∥_ ≈ 137 × 10^−4^ cm^−1^). As the pH increased to 6.0–9.0, two sets of hyperfine splitting (component I and II) were clearly superposed in the spectra.^22^ Component I predominated initially, whereas the relative proportion of component II gradually increased with rising pH. At alkaline pH (9.0–10.0), the more stable component II became the dominant species (Fig. 2B). This reversible redistribution suggested an equilibrium between two coexisting Cu(ii) coordination modes, which shifted in response to changes in proton concentration.

Overall, despite the differences in their specific transitional behaviors, both peptide systems exhibit a highly consistent macroscopic trend as pH increases: the quartet hyperfine splitting in the parallel region progressively shifts to higher magnetic fields, accompanied by a decrease in *g*_∥_ and a concurrent increase in *A*_∥_ (Table S2). Mechanistically, this unified pH-dependent evolution can be explained by changes in the local ligand field. Increasing pH induces the sequential deprotonation of peptide functional groups.^23^ These deprotonated nitrogen atoms act as strong-field ligands, progressively displacing the weaker oxygen donors in the equatorial plane.^16,24^ Compared to oxygen, nitrogen donors form stronger and more covalent in-plane σ-bonds with the Cu(ii) ion. This enhanced equatorial bonding effectively constrains the complex into a more rigid and planar arrangement. The increased covalency promotes delocalization of the unpaired electron, thereby reducing the *g*_∥_ values. Concurrently, the tighter planar geometry strengthens the interaction between the unpaired electron and the copper nucleus, leading to an increase in *A*_∥_.^14,25^

Thus, increasing pH progressively unlocks strong-field nitrogen donors, driving a geometric and electronic transformation from a weakly coordinated, low-covalency oxygen-rich mode to a strongly coordinated, highly covalent nitrogen-rich mode. This pH-induced transformation from a weak to a strong coordination mode may, in turn, modulate the redox properties of the Cu(ii) centre.

**Fig. 3:**
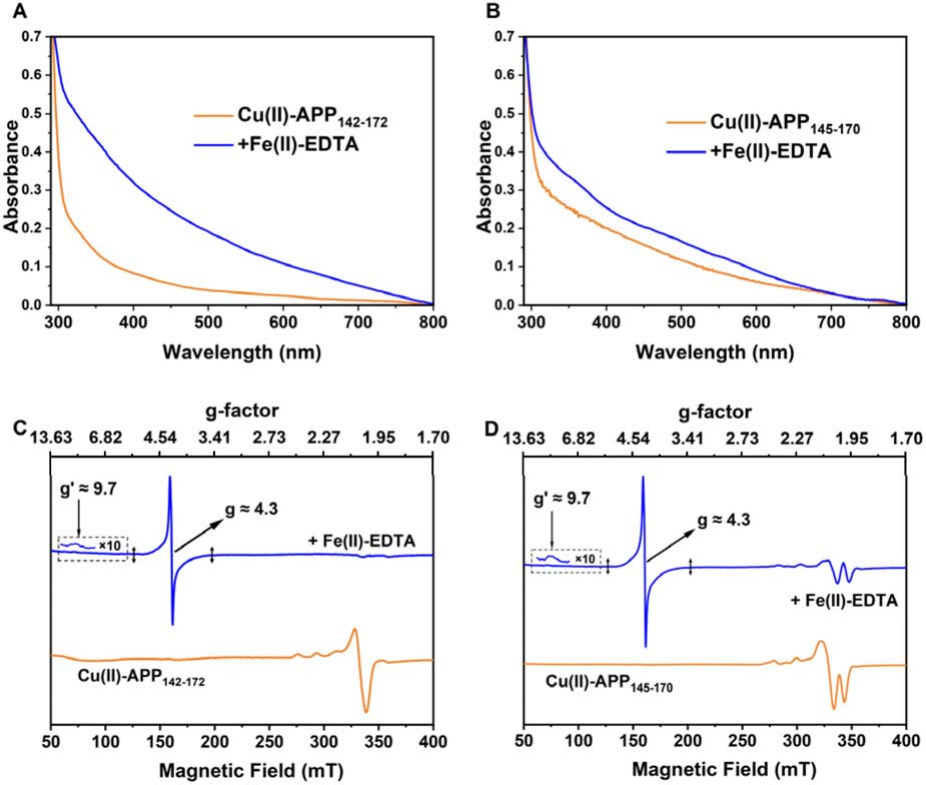
Fe(ii) oxidation by Cu(ii)–peptide complexes under anaerobic conditions. (A and B) Spectra of Cu(ii)–APP_142–172_ and Cu(ii)–APP_145–170_ before (orange) and after (blue) addition of Fe(ii)–EDTA. (C and D) X-band CW-EPR spectra of Cu(ii)–APP_142–172_ and Cu(ii)–APP_145–170_ before (orange) and after (blue) addition of excess Fe(ii)–EDTA. After Fe(ii) addition, a broad feature at *g* ≈ 4.3 (≈700 G) and a weak feature near *g*′ ≈ 9.7 are observed, consistent with high-spin (*S* = 5/2) Fe(iii) in rhombic, strong-ZFS environments and indicative of substantial *D*-strain.

To assess the impact of coordination mode on the redox behaviours of Cu(ii)–peptide complexes, EPR and UV-Vis spectroscopy were employed to investigate their reactions with Fe(ii). To further evaluate the redox properties of these complexes towards physiologically relevant Fe(ii) species, two Fe(ii) chelators, Fe(ii)–EDTA and Fe(ii)–citrate, were used to model Fe(ii) coordination environments in ferroxidases. These Fe(ii) complexes were added to solutions of Cu(ii)–APP_142–172_ and Cu(ii)–APP_145–170_, and the mixtures were incubated for 30 min under anaerobic conditions. UV-Vis spectra revealed a pronounced increase in absorbance at 315 nm, indicating the formation of Fe(iii) species (Fig. 3A and B).^26^ Upon addition of 10 equivalents of Fe(ii)–EDTA, EPR monitoring revealed an increased signal at *g* ≈ 4.3 in both systems, consistent with high-spin (*S* = 5/2) Fe(iii) in a low-symmetry environment (Fig. 3C, D, and S8). Under X-band conditions (|*D*| ≫ *hν*), this feature arises from transitions within the lowest Kramers doublet of rhombic Fe(iii).^21,27,28^

Simultaneously, the Cu(ii) signal in the Cu(ii)–APP_142–172_ complex disappeared completely, indicating reduction from EPR-active Cu(ii) to EPR-silent Cu(i) (Fig. 3C). In contrast, the two components of Cu(ii)–APP_145–170_ complex displayed distinct spectral behaviours upon Fe(ii) addition: the signal intensity of component I became undetectable, whereas component II remained observable (Fig. 3D). This differential response demonstrated that the two Cu(ii) coordination geometries in Cu(ii)–APP_145–170_ had different susceptibilities to reduction during Fe(ii) oxidation.

These different spectral responses suggest that ligand identity and electronic structure may contribute to the differing redox susceptibilities of component I and component II. Component I, with its mixed N/O coordination, provides the structural plasticity required to accommodate geometric rearrangement of the Cu(ii)/Cu(i) redox couple,^25^ thereby driving reversible redox cycling coupled to Fe(ii) oxidation. By contrast, the nitrogen-rich coordination of component II provides strong σ-donor interactions and enhanced covalency that significantly stabilize the Cu(ii) state.^29^ Consequently, the reduction potential shifts negatively, rendering component II redox-inactive under physiological conditions.^30,31^

**Fig. 4:**
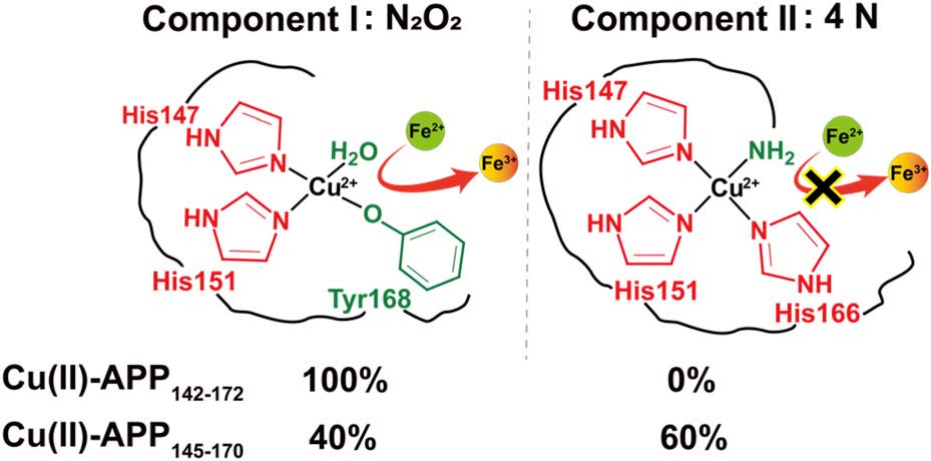
Proposed coordination modes of component I and component II.

In this study, we elucidated how subtle sequence variations in the CuBD core region of APP influenced copper coordination modes and, consequently, determined the resulting redox behaviours. EPR spectroscopy revealed that Cu(ii)–APP_142–172_ adopted a N_2_O_2_ coordination mode at physiological pH, whereas Cu(ii)–APP_145–170_ existed in two distinct coordination modes: the same N_2_O_2_ form (component I) observed for Cu(ii)–APP_142–172_, and a nitrogen-rich 4N form (component II). Given that component I was observed in both copper–peptide complexes, with spin Hamiltonian parameters closely matching those of the Cu(ii)–CuBD complex under identical experimental conditions.^8^ Its coordination sphere likely comprised a typical N_2_O_2_ environment formed by His147, His151, Tyr168, and water molecules (Fig. 4). The distinctive coordination behaviour of APP_145–170_ can be attributed to the selective release of local structural constraints. Specifically, the shorter fragment length may induce relaxation or local rearrangement of the β-sheet region containing His166, thereby increasing its conformational mobility. This allowed His166 to approach the N-terminus and the nearby α-helical histidine cluster and thus to be recruited into the same coordination sphere. Based on sequence analysis and literature comparisons, we proposed that the 4N coordination of component II involved the imidazole nitrogens of His147, His151, and His166, as well as the exposed N-terminal amino nitrogen (Fig. 4). As assignments based on the Peisach–Blumberg plot were inherently empirical, the N_2_O_2_ and 4N classifications proposed here should be considered tentative and interpreted with appropriate caution.

## 3. Conclusions

In summary, our results reveal that subtle sequence variations among APP CuBD-derived fragments alter copper coordination modes and thereby modulate redox properties. Specifically, Cu(ii)–APP_142–172_ and component I of Cu(ii)–APP_145–170_ predominantly adopt N_2_O_2_ coordination mode, whereas component II of the shorter fragment adopts 4N coordination mode. Notably, the relative populations of these two coordination modes show distinct pH dependence. Critically, these structural differences lead to distinct functional properties: the N_2_O_2_ coordination readily promotes Fe(ii) oxidation, while the 4N coordination, with its stronger ligand field, remains redox-inactive. Taken together, these findings establish a direct sequence–structure–function relationship in APP CuBD fragments. However, this study was conducted on short, soluble peptide fragments of the APP CuBD in aqueous buffer under anaerobic conditions, with EPR spectra acquired at 80 K. In the physiological environment, the CuBD is embedded within full-length, transmembrane APP or its shed soluble ectodomain (sAPP),^24^ where membrane anchoring, protein–protein interactions, and cellular reductants may further modulate coordination geometry and redox behaviour. Furthermore, in Alzheimer's disease, copper is highly enriched in extracellular senile plaques composed of aggregated Aβ peptides. Within these dense fibrillar deposits, where solvent and O_2_ diffusion are restricted, Cu(ii) can be rapidly reduced to EPR-silent Cu(i), and the generation of catalytic reactive oxygen species (ROS) remains dynamically active at physiological temperature (37 °C).^32^ Consequently, the coordination modes and redox activities reported here likely represent only a subset of the complex behaviours of CuBD in cells or in the crowded microenvironment of amyloid plaques. Nevertheless, the demonstration that peptide length can switch coordination between redox-active N_2_O_2_ and redox-silent 4N species suggests that controlling peptide length or conformation could serve as a strategy to probe or modulate copper-driven redox chemistry. This finding provides a preliminary molecular rationale for metal-targeted therapies in Alzheimer's disease, although its physiological relevance remains to be validated in cellular and *in vivo* models.

## 4. Experimental section

### 4.1 Materials and reagents

All chemicals were of analytical (ACS) grade unless otherwise noted and were used without further purification. The reagents employed in this study included glycerol, hydrochloric acid (HCl), sodium hydroxide (NaOH), sodium chloride (NaCl), copper(ii) sulfate pentahydrate (CuSO_4_·5H_2_O), ferrous ammonium sulfate hexahydrate ((NH_4_)_2_Fe(SO_4_)_2_·6H_2_O), EDTA disodium salt dihydrate (Na_2_EDTA·2H_2_O), sodium citrate, anhydrous potassium dihydrogen phosphate (KH_2_PO_4_), and potassium hydrogen phosphate (K_2_HPO_4_).

### 4.2 Preparation of peptide samples

The peptides APP_142–172_ (DVCETHLHWHTVAKETCSEKSTNLHDYGMLL) and APP_145–170_ (ETHLHWHTVAKETCSEKSTNLHDYGM), in lyophilized form, were obtained from QYAOBIO and verified by reversed-phase high performance liquid chromatography (RP-HPLC) and mass spectrometry (Fig. S1–S4). Both peptides possessed a free N-terminal amine and an amidated C-terminus.

Stock solutions (2.0 mM) were prepared by dissolving the samples in ultrapure water. For spectroscopic experiments, aliquots of the 2.0 mM stock solution were diluted with KPi buffer (25 mM, pH 7.4) to a final concentration of 0.5 mM, and pH adjustments were made with 0.10 M HCl or 0.10 M NaOH as required.^33^

Cu(ii)–APP_142–172_ and Cu(ii)–APP_145–170_ complexes were prepared by mixing the peptide solutions with 1.25 equivalents of CuSO_4_ and stirring at 4 °C for 1 h. Unbound CuSO_4_ was removed using a PD-10 desalting column (Sephadex G-25, Cytiva) pre-equilibrated with the same anaerobic buffer used for the experiments.

Samples prepared for CW-EPR contained 500 µM peptide in 25 mM KPi buffer (pH 7.4) and were supplemented with glycerol (final concentration 10% v/v) as a cryoprotectant before being transferred into EPR tubes (707-SQ-250M, 4 mm OD, Wilmad LabGlass). Each CW-EPR sample volume was 100 µL. For W-band pulsed experiments, samples were prepared by dissolving the peptide–copper complexes in D_2_O, loading 2 µL into quartz capillaries, and sealing the capillaries with wax. All CW-EPR samples were flash-frozen and stored in liquid nitrogen prior to measurement.

All subsequent experiments were performed in an anaerobic chamber (Coy Laboratory Products, Grass Lake, MI, USA). Buffers and experimental materials were thoroughly degassed and equilibrated in the anaerobic chamber for at least 24 hours prior to use.

### 4.3 EPR measurements

Low-temperature continuous-wave (CW) X-band EPR spectra were recorded on a Bruker X-band EMXplus 10/12 spectrometer equipped with an Oxford ESR-910 helium flow cryostat and ITC-503 temperature controller (Oxford Instruments Ltd, Oxfordshire, UK). Data were collected using a cylindrical resonator (ER4119HS E011). The magnetic field was calibrated using a DPPH standard (*g* = 2.0036) before each series of measurements. The experimental conditions were as follows: temperature, 80 K; modulation amplitude, 5 G; microwave power, 2 mW; microwave frequency, 9.445 GHz; and time constant, 163.84 ms. To achieve a good signal-to-noise ratio, typically 10 scans were accumulated for each sample.

All EPR spectra were background-subtracted using KPi buffer blanks and subsequently baseline-corrected with a third-order polynomial using the *WinEPR Processing* software. The EPR spectra were simulated using EasySpin.

W-band pulsed EPR experiments were performed on a CIQTEK W900 spectrometer (CIQTEK (Hefei) Technology Co., Ltd, Hefei, China) equipped with a closed-cycle, cryogen-free variable-temperature system.

Davies ENDOR spectra were recorded at 10 K. The static magnetic field was set to 32 556 G, corresponding to the maximum echo intensity in the field-swept echo-detected EPR spectrum. The microwave pulse sequence employed a Davies ENDOR scheme with pulse lengths of 280–140–280 ns, with an RF pulse of 18 µs duration inserted between the second and third microwave pulses. The two microwave attenuation channels were set to 1 dB (LowAttenuation) and 29 dB (HighAttenuation), corresponding to an incident microwave power of approximately 1.05 mW at the resonator. The RF channel was operated at a power of 10.0 dBm. The RF frequency was swept from 0.1 to 150.1 MHz over 600 data points. A repetition time of 2 ms and 100 shots per point were used, and spectra were accumulated over 50 sweeps.

### 4.4 Ferroxidase assay

Fe(ii)–EDTA and Fe(ii)–citrate solutions were prepared by mixing (NH_4_)_2_Fe(SO_4_)_2_·6H_2_O with EDTA disodium salt or sodium citrate at 1 : 1 molar ratio, followed by dilution to a final concentration of 25 mM in an anaerobic chamber. The stock solutions were stirred under anoxic conditions for 30 min prior to use. All subsequent dilutions were made in KPi buffer (pH 7.4) that had been thoroughly degassed and equilibrated in the anaerobic chamber.

Reaction mixtures were prepared by rapidly mixing 500 µM Cu(ii)–peptide with varying concentrations of labile Fe(ii), Fe(ii)–EDTA, or Fe(ii)–citrate in KPi buffer (pH 7.4). 300 µL of each reaction mixture were transferred to UV-transparent quartz cuvettes (2 mm optical path length, nominal volume 0.7 mL, screw-cap sealed) and the absorption spectra were recorded at 25 °C using a UV-Vis spectrophotometer (UV670, Shanghai Meipada Instrument Co., Ltd). Glycerol was then added to the remaining reaction mixture to a final concentration of 10% v/v, and 100 µL aliquots were withdrawn for low-temperature EPR measurements.

## Conflicts of interest

There are no conflicts to declare.

## Supplementary Material

RA-016-D6RA00647G-s001

## Data Availability

All data supporting the findings of this study are available within the article and its supplementary information (SI). Supplementary information: additional EPR spectra, spectral simulations, and figures and tables. See DOI: https://doi.org/10.1039/d6ra00647g.
