# Supplementary material for: EPR spectroscopy reveals different Cu(ii) coordination in APP142–172 and APP145–170 peptide fragments of amyloid precursor protein
Source: RSC Adv. 2026 Mar 19;16(17):15504–9. doi: 10.1039/d6ra00647g (PMC13000901; doi:10.1039/d6ra00647g)
Supplement: RA-016-D6RA00647G-s001 [file RA-016-D6RA00647G-s001.pdf]

Supplementary Information (SI) for RSC Advances.
This journal is © The Royal Society of Chemistry 2026

Supplementary Information

EPR Spectroscopy Reveals Different Cu(II) Coordination in $APP_{142–172}$ and $APP_{145–170}$ Peptide Fragments of Amyloid Precursor Protein

Liya Xu[a, b], Jian Kuang[b], Aokun Liu[c], Lianghuan Liao[c], Lu Yu*[b] and Changlin Tian*[a,c]

[a.] High Magnetic Field Laboratory, Hefei Institutes of Physical Science, Chinese Academy of Sciences, Hefei, Anhui 230031, China.

[b.] Department of Environmental Science and Engineering, University of Science and Technology of China, Hefei, Anhui 230026, China

[c.] School of Chemistry and Chemical Engineering, Zhang jiang Institute for Advanced Sciences, Shanghai Jiao Tong University, Shanghai 201203, China.

Corresponding Author

*E-mail: luyuesr@ustc.edu.cn

cltian@sjtu.edu.cn

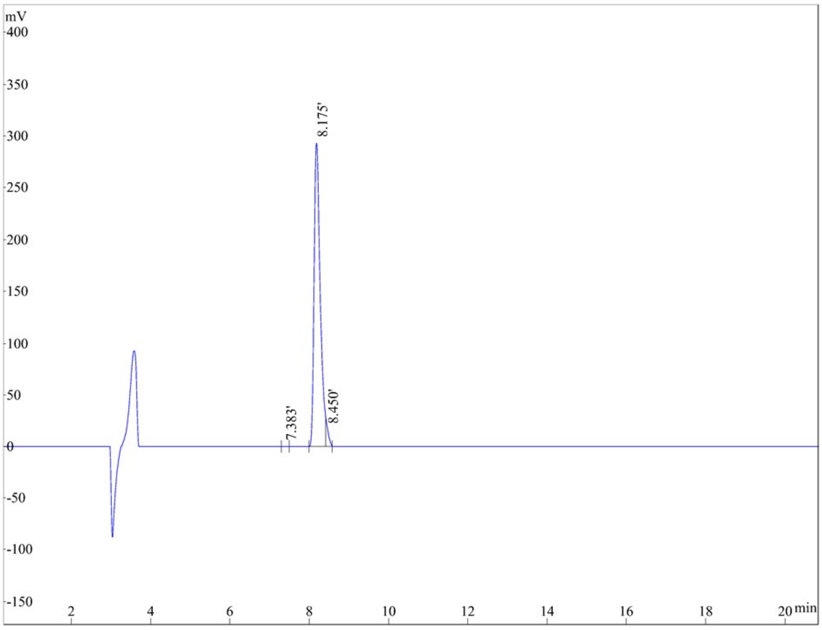

Fig. S1 RP-HPLC chromatogram of $APP_{142\text{-}172}$. Chromatography was performed on a C18 column (4.6 × 250 mm, 5 µm) using a linear gradient of acetonitrile/water containing 0.1% TFA, monitored at 220 nm. The peptide eluted as a major peak at 8.18 min with a purity of 96.8%.

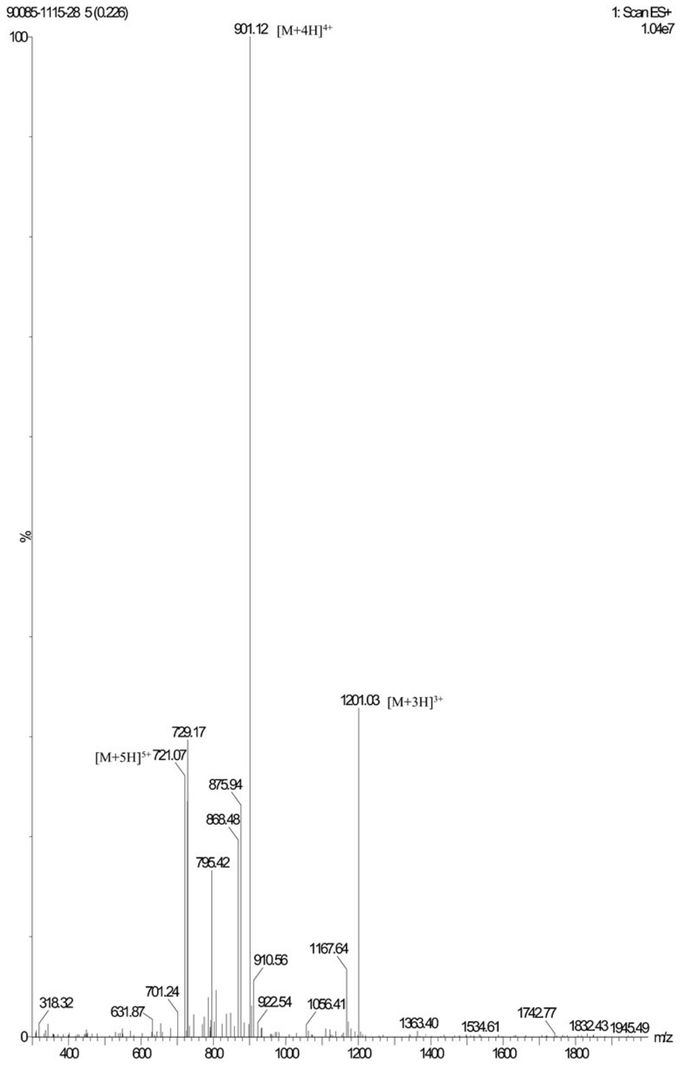

Fig. S2 ESI-MS spectrum of peptide $APP_{142\text{-}172}$. The observed multiply charged ions are consistent with the calculated molecular weight of the peptide (3599.0 Da).

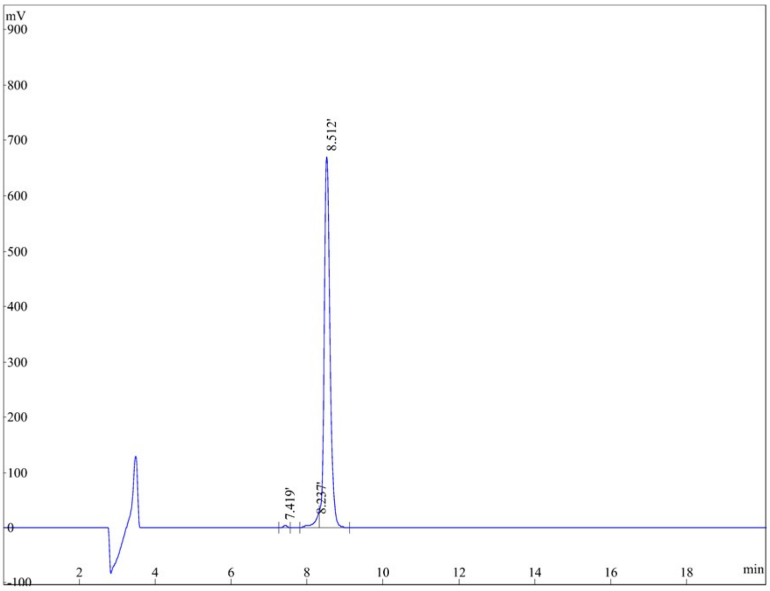

Fig. S3 RP-HPLC chromatogram of $APP_{145\text{-}170}$. Analysis was carried out on a C18 column with acetonitrile/water (0.1% TFA) as the mobile phase and UV detection at 220 nm. The main peak eluted at 8.51 min, corresponding to a purity of 95.6%.

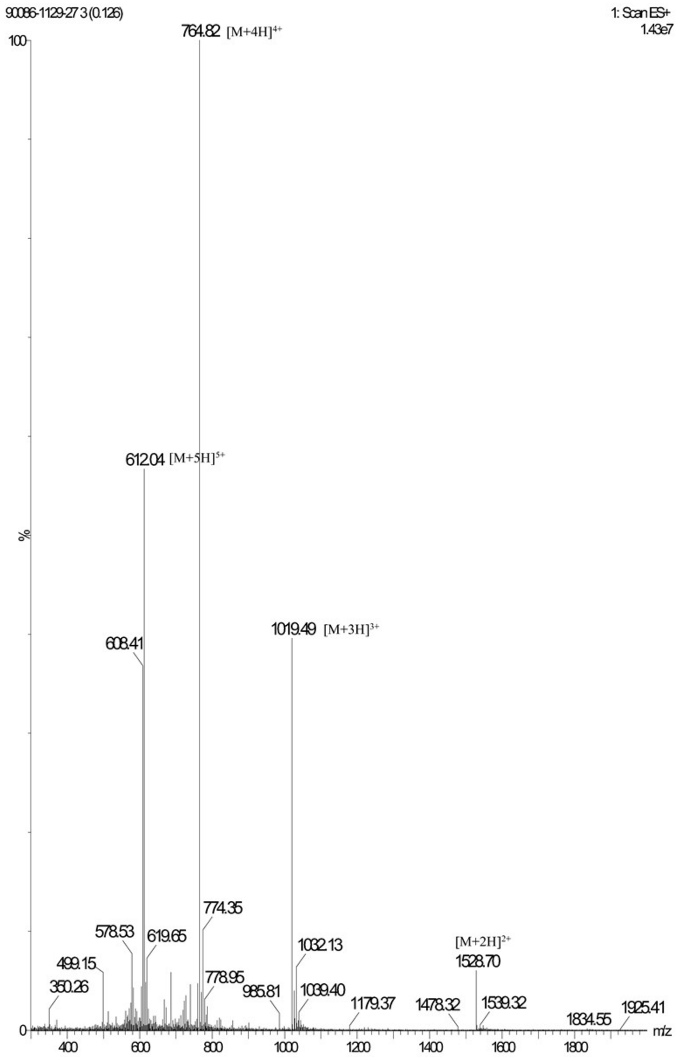

Fig. S4 ESI-MS spectrum of peptide $APP_{145\text{-}170}$. The experimental mass spectrum shows the expected charge-state distribution, confirming the molecular weight of the peptide (3055.4 Da).

Parameter	Cu(II)–APP$_{142–172}$	Cu(II)–APP$_{145–170}$
**$g_{\parallel}$**	2.2725	2.2725
		2.1850
**$g_{\perp}$**	2.0553	2.0553
		2.0350
**$A_{\parallel}$ (MHz)**	515	515
		600
**$A_{\perp}$ (MHz)**	35	35
		80
**g-strain(x/y/z)**	0.01/0.01/0.03	0.01/0.01/0.03
		0.01/0.01/0.03
**A-strain(x/y/z)**	10/10/90	10/10/90
		20/20/80
**g-AstrainCorr**	-1	-1
		-1
**Proportion**	1	0.4
		0.6
**$f = g_{\parallel} / A_{\parallel}$ (cm)**	132.12	132.12
		109.25

Table S1 Summary of simulation parameters obtained from EasySpin fitting of the EPR spectra. The ratio of $g_{\parallel}/A_{\parallel}$, denoted as f, can be considered as a measure of structural distortion and deviation from ideal geometry. The f value in the range of 110–120 cm is characteristic for Cu(II) centers with planar geometry, whereas its increase to approximately 150 cm indicates a slight to moderate distortion from planar symmetry.

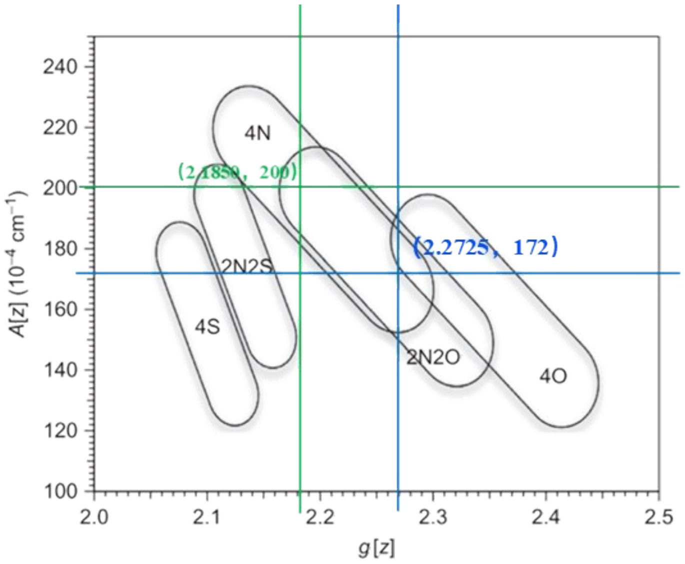

Fig. S5 Peisach-Blumberg correlation plot of the copper(II) EPR parameters, $g_{\parallel}$ versus $A_{\parallel}$. The blue traces correspond to component I, whose parameters fall within the N_2O_2 region, indicating a mixed nitrogen-oxygen coordination sphere. The green traces represent component II, located near the 4N region, consistent with a nitrogen-rich ligand environment.

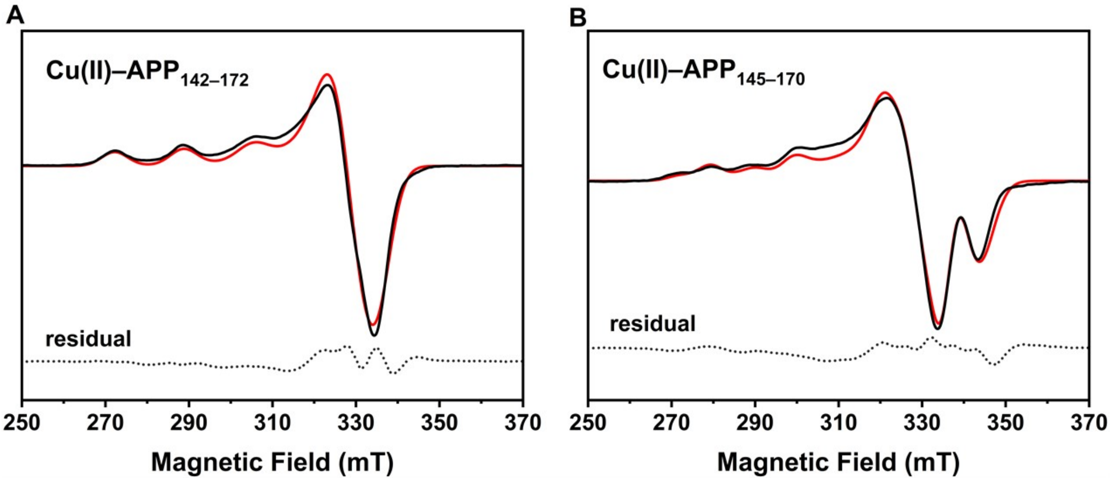

Fig. S6 Residual spectra (Experimental spectra subtracted by Simulated spectra) for (A) Cu(II)–APP$_{142–172}$ and (B) Cu(II)–APP$_{145–170}$, demonstrating the quality of the fits shown in Fig. 1.

pH	Cu(II)–$APP_{142–172}$ $g_{\parallel}$	Cu(II)–$APP_{142–172}$ $A_{\parallel}$ (10^{-4} cm^{-1})	Cu(II)–$APP_{145–170}$ $g_{\parallel}$	Cu(II)–$APP_{145–170}$ $A_{\parallel}$ (10^{-4} cm^{-1})
**5.0**	2.407	135	2.406	137
**6.0**	2.261	182	2.268	182
			2.171	213
**7.0**	2.260	184	2.258	181
			2.177	209
**8.0**	2.264	177	2.275	183
			2.182	207
**9.0**	2.261	179	2.279	185
			2.180	207
**10.0**	2.263	179	2.183	204

Table. S2 pH-dependent EPR parameters ($g_{\parallel}$ and $A_{\parallel}$) for Cu(II)–$APP_{142–172}$ and Cu(II)–$APP_{145–170}$ complexes. For Cu(II)–$APP_{145–170}$, values for both Component I and II are listed where applicable.

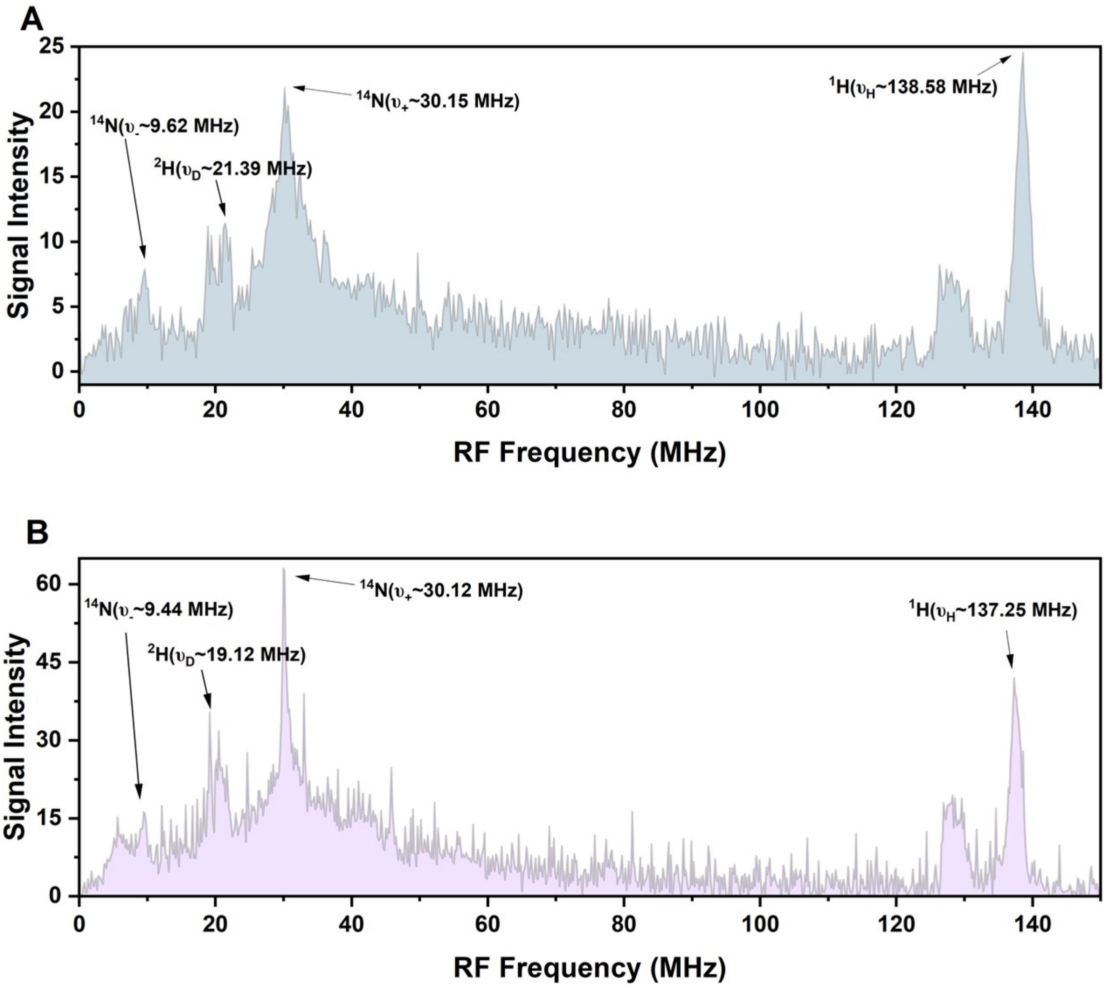

Fig. S7 Preliminary W-band Davies ENDOR spectra of Cu(II)-APP$_{142\text{-}172}$ (A) and Cu(II)-APP$_{145\text{-}170}$ (B) recorded at 10 K. The spectra were obtained at the magnetic field setting corresponding to the maximum of the electron spin echo ($g_{\perp}$ region).

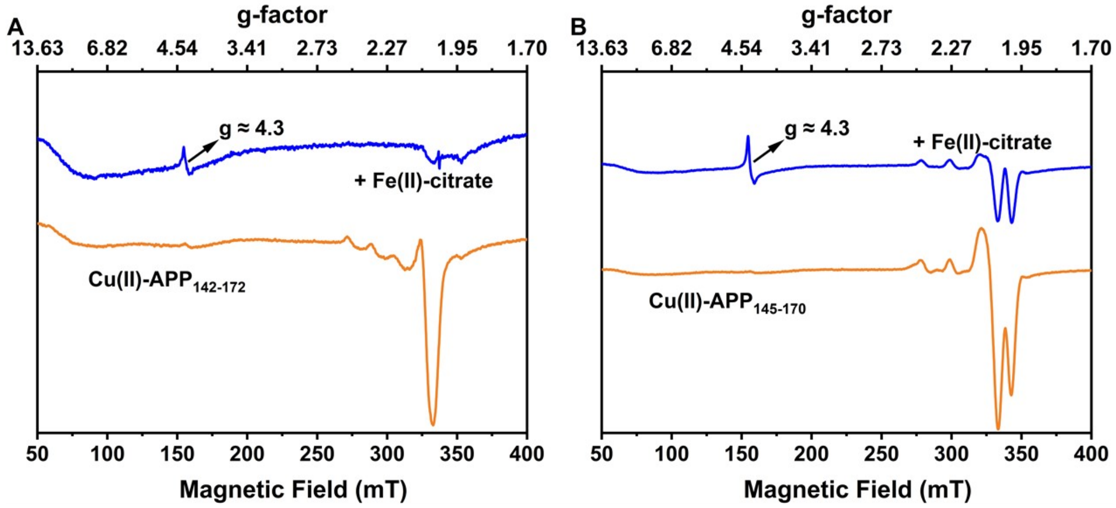

Fig. S8 Fe(II)-citrate oxidation by Cu(II)–peptide complexes monitored by EPR spectroscopy under anaerobic conditions. (A) Cu(II)–$APP_{142–172}$ before (orange) and after (blue) addition of excess Fe(II)-citrate. The Cu(II) signal is effectively eliminated upon Fe(II)-citrate addition, consistent with reduction to EPR-silent Cu(I). The minor feature near g ≈ 2.0 in the blue trace of panel A originates from a light-induced resonator baseline distortion and is not sample-derived. (B) Cu(II)–$APP_{145–170}$ before (orange) and after (blue) Fe(II)-citrate addition. A residual Cu(II) contribution persists, indicating incomplete reduction in this system. The signal at g ≈ 4.3 in both panels is characteristic of high-spin Fe(III).
